# Genomic Characterization, Formulation and Efficacy in Planta of a *Siphoviridae* and *Podoviridae* Protection Cocktail against the Bacterial Plant Pathogens *Pectobacterium* spp.

**DOI:** 10.3390/v12020150

**Published:** 2020-01-28

**Authors:** Maja A. Zaczek-Moczydłowska, Gillian K. Young, James Trudgett, Colin C. Fleming, Katrina Campbell, Richard O’Hanlon

**Affiliations:** 1 Institute for Global Food Security, School of Biological Sciences, Queen’s University, Belfast BT9 5DL, UK; katrina.campbell@qub.ac.uk; 2 Sustainable Agri-Food Sciences Division, Agri-Food and Biosciences Institute, Belfast, BT9 5PX UK; Gillian.Young@afbini.gov.uk (G.K.Y.); Colin.Fleming@afbini.gov.uk (C.C.F.);; 3 Veterinary Sciences Division, Agri-Food and Biosciences Institute, Stormont, Belfast BT4 3SD, UK; James.Trudgett@afbini.gov.uk

**Keywords:** bacteriophages, phage cocktail, *Pectobacteriacea*, biocontrol

## Abstract

In the face of global human population increases, there is a need for efficacious integrated pest management strategies to improve agricultural production and increase sustainable food production. To counteract significant food loses in crop production, novel, safe and efficacious measures should be tested against bacterial pathogens. *Pectobacteriaceae* species are one of the causative agents of the bacterial rot of onions ultimately leading to crop losses due to ineffective control measures against these pathogens. Therefore, the aim of this study was to isolate and characterize bacteriophages which could be formulated in a cocktail and implemented *in planta* under natural environmental conditions. Transmission electron microscopy (TEM) and genome analysis revealed *Siphoviridae* and *Podoviridae* family bacteriophages. To test the protective effect of a formulated phage cocktail against soft rot disease, three years of field trials were performed, using three different methods of treatment application. This is the first study to show the application of a phage cocktail containing *Podoviridae* and *Siphoviridae* bacteriophages capable of protecting onions against soft rot in field conditions.

## 1. Introduction

Despite recent advances in food shelf-life extension technologies, improved varieties of crops and more efficient food distribution systems, food waste has been estimated at 1.3 billion tons per year, which is equal to one-third of all food produced for human consumption [1]. Food is wasted throughout the food supply chain, with significant loss and waste during agricultural production in economically important crops such as potato [1]. Plant diseases, caused by a wide range of pests (including pathogens), are among the largest causes of global crop losses, estimated to be 27–42% in production systems [2]. The production of onions (*Allium cepa* L.) is of high economic importance, representing 12%, by mass, of global production of vegetables (93.16 million tons). The most intensive production of onions occurs in Asia with 62.2%, Africa at 12.5% and Europe at 10.6%, with the global gross production value evaluated at up to US $42.1 million [3]. Appropriate control measures for onions against bacterial rot disease are difficult to achieve, given that at least 26 different fungi and bacteria are associated with this rot [4]. Onion rot may ultimately cause significant post–harvest losses, with disease beginning in the field and developing further during transit and storage [5]. Thus far, soft rot disease management mainly consists of preventative measures based on good plant handling hygiene, with no biological or chemical control agents available commercially [6].

The most destructive pathogens reported to cause disease in onions are *Xanthomonas retroflexus* (leaf blight), *Pseudomonas viridiflava* and *Xanthomonas* spp. (bacterial streak and bulb rot) [5]. A diverse range of bacteria causing soft rot of onions have been reported, including *Pseudomonas* spp. (e.g., *Pseudomonas marginalis, Pseudomonas allicola,* and *Pseudomonas cepacia*) [5,7], *Pectobacterium* spp. (*Pectobacterium carotovorum* subsp. *carotovorum (P. c.* subsp. *carotovorum)* and *Pectobacterium carotovorum* subsp. *odoriferum*) [8], and *Dickeya* spp. (e.g. *Dickeya chrysanthemi*, *Dickeya fangzhongdai*) [9,10]. 

Previous phage therapy trials in greenhouses and field conditions have been carried out against soft rot *Pectobacteriaceae* (SRP) of potato, including *Dickeya* sp. [11] and *Pectobacterium* spp. [12,13]. Bacteriophages have been evaluated as phage therapy in agriculture to combat threats related to pathogenic bacterial species such *Erwinia amylovora*, *Xantomonas campestris* or *Dickeya* spp. [14], with one report describing attempts to control onion blight disease of onions (causal agent *Xanthomonas* sp.) using a bacteriophage formulation [15]. Phage cocktails used against soft rot have been designed based on lytic activity and host range, using methods such as overlay, spotting assays, killing curves and semi—in planta experiments [16,17]. No standardized method exists for the process of screening microorganisms as potential biocontrol candidates for plant protection products, though general guidance is available for biocidal purposes [18]. Caution should be taken when assuming correlation between in vitro and field efficacy results as this process should be based directly on the pathosystem [2,19].

This study focused on the isolation and genomic characterization of prospective phages to target a known pathogen of onion: *P. c*. subsp. *carotovorum*. This is the first study providing the characterization of broad host range *Podoviridae* and *Siphoviridae* family bacteriophages infecting *P. c*. subsp. *carotovorum* and *Pantoea* spp. This study also provides an evaluation of a phage cocktail to protect onions against natural soft rot infection in three years of field trials using three different methods of application.

## 2. Materials and Methods

### 2.1. Bacterial Isolates and Media

Bacterial strains used in this study were isolated from soft rot tissue of onions growing in Northern Ireland and characterized to be *Pectobacterium* spp. Two isolated strains were identified as *Pantoea* spp. (Table S1). Bacteria were grown on crystal violet pectate media (CVP), with modifications reported previously [20]. Selected bacterial cultures were grown at 25 °C for 24–48 h and were purified twice on nutrient agar (NA) CM0003 (Oxoid, Basingstoke, UK). Bacterial DNA was extracted using Maxwell^®^ 16 cell LEV DNA Purification kit (Promega, Madison, WI, USA) following the manufacturer’s instructions and purified for 30–45 min using a Maxwell DNA Magnetic Particle Processor MX 3031 (Promega, Madison, WI, USA) using the cell purification program. DNA extracts were quantified using a NanoDrop 2000 (Thermo Scientific, Waltham, MA, USA), and the concentrations of bacterial DNA extracts adjusted to between 10–20 ng/µL. Purified bacterial DNA was stored at −20 °C until further analysis. Obtained bacterial DNA extracts were confirmed using real–time polymerase chain reaction (PCR), conventional PCR and/or *recA* sequencing as *Pectobacterium* spp., *Pectobacterium atrosepticum* (*P. atrosepticum*), *P. c.* subsp. *carotovorum* and *Pantoea* spp. (Table S1) using PCR amplification conditions and sequenced as reported previously [20]. 

To produce the liquid bacterial culture used for enrichment of bacteriophages, pure bacterial colonies were harvested and inoculated in nutrient broth (NB) containing peptone (1 g, Sigma Aldrich, St. Louis, MO, USA), yeast extract (0.5 g, Oxoid, Basingstoke, UK), NaCl (0.25 g, Thermo Fisher Scientific, Waltham, MA, USA), K_2_HPO_4_ (0.8 g, Thermo Fisher Scientific, Waltham, MA, USA) per 100 mL, for 12–24 h at 25 °C with 200 rpm agitation and adjusted to ca. 10^8^ CFU/mL in NB (approx. OD A_600_ = 0.2).

### 2.2. Isolation, Purification and Enrichment of Bacteriophages

Bacteriophages were isolated from potato wastewater samples obtained from the Department of Agriculture, Environment and Rural Affairs Northern Ireland (DAERA) by filtration using a filtration unit and receiver flask Stericup™ Millipore Express TM^®^ Plus 0.22 μm (Merck, Darmstadt, Germany) connected to a general purpose vacuum pump (KNF Neuberger, ultimate vacuum 100 Mbar, flow rate 15 L/min). The volume of 40 mL of processing water sample was poured into the Steritop™ filter (Merck, Darmstadt, Germany) to obtain bacteriophage filtrate in the receiver flask.

For enrichment of bacteriophages, a volume of 5 mL of sterile 10× NB containing peptone (20 g, Sigma Aldrich, St. Louis, MO, USA), yeast extract (10 g, Oxoid, Basingstoke, UK), NaCl (5 g, Thermo Fisher Scientific, Waltham, MA, USA) and K_2_HPO_4_ (16 g, Thermo Fisher Scientific, Waltham, MA, USA) per 200 mL was added to the Stericup™ Millipore Express TM^®^ Plus filter receiver flask (Merck, Darmstadt, Germany) containing the filtered potato wastewater, followed by equal volumes (2.5 mL) of each bacterial liquid cultures (*ca*. 10^8^ CFU/mL) (OD A_600_ = 0.2) (Table S1). The resulting solution (bacteriophage and bacteria) was incubated at 25 °C with 200 rpm agitation for 12–24 h. Following that, an aliquot of 10 mL of the solution was transferred into a centrifuge tube and centrifuged at (2000 rpm, 5 °C) for 5 min. The supernatant which contained bacteriophages was filtered using a 10 mL syringe barrel fitted with a 0.22 μm filter Millex^®^ GV filter unit (Merck, Darmstadt, Germany). This bacteriophage filtrate was stored at 4 °C until use. The 100 μL of filtrate was added to 900 μL of sterile phosphate–buffered saline (PBS) buffer containing NaCl (1.6 g, Sigma Aldrich, St. Louis, MO, USA), KCl (0.04 g, Thermo Fisher Scientific, Waltham, MA, USA), K_2_HPO_4_ (0.22 g, Sigma Aldrich, St. Louis, MO, USA) and KH_2_PO_4_ (0.04 g, Thermo Fisher Scientific, Waltham, MA, USA) per 100 mL, pH 7.4. Ten–fold serial dilutions were made in PBS buffer pH 7.4 (Neat, 10^−1^, 10^−2^, 10^−3^, 10^−4^) and subjected to plaque formation using the double layer agar method. 

For plaque formation using the double layer agar method, the filtered potato wastewater was combined with equal volumes (250 μL) of each liquid bacterial culture (Table S1) and 100 μL of each bacteriophage in a ten—fold dilution and then incubated at 25 °C for 20 min to allow the phage to adsorb to the bacteria. The 3–5 mL of top agarose (TA) (37 °C) containing peptone (10 g, Sigma Aldrich, St. Louis, MO, USA), yeast extract (5 g, Oxoid, Basingstoke, UK), NaCl (2.5g, Thermo Fisher Scientific, Waltham, MA, USA), K_2_HPO_4_ (8 g, Thermo Fisher Scientific, Waltham, MA, USA) and low gelling agarose (7.5 g, Sigma Aldrich, St. Louis, MO, USA) per litre was added to a 30 mL Sterilin® universal container (Thermo Fisher Scientific, Waltham, MA, USA). The container was capped quickly and mixed gently. The mixture was immediately poured onto the NA plates (CM0003, Oxoid, Basingstoke, UK) and left until the agarose solidified, and then incubated at 25 °C for 24 h. 

Bacteriophages were purified through picking plaques obtained on NA plates (CM0003, Oxoid, Basingstoke, UK), using sterile pipette tips and eluting in 900 μL of PBS buffer pH 7.4, following ten—fold dilutions. This step was repeated 3–5 times. To obtain bacteriophage lysate, double layer plates with phage plaques were subsequently re-suspended by adding 4 mL of PBS buffer at pH 7.4. After 3 h, harvested lysate was filter–sterilized using a 10 mL syringe barrel fitted (Thermo Fisher Scientific, Waltham, MA, USA ) with a 0.22 μm filter Millex^®^ GV filter unit (Merck, Darmstadt, Germany) and maintained in a 30 mL Sterilin^®^ universal container (Thermo Fisher Scientific, Waltham, MA, USA) at 4 °C. To determine the concentration of bacteriophage lysate, the volume of 10 μL of lysate was added to 990 μL of PBS buffer pH 7.4. Ten–fold serial dilutions were made in PBS buffer pH 7.4 and subjected to plaque formation for 24 h using the double layer agar method. The concentration of each bacteriophage lysate was expressed as plaque forming units (PFU/mL).

### 2.3. TEM

Bacteriophage lysate (*ca*. 10^8^–10^14^ PFU/mL) was concentrated and purified by centrifugation with modifications to the method previously described [21]. Modifications involved centrifugation at (30,000× *g*, 4 °C) for each purification step and washing with 500 μL 1 M ammonium acetate, pH 7.4 (Thermo Fisher Scientific, Waltham, MA, USA). The copper grids were placed on 10 µL of pellet containing bacteriophages for 15 min to adsorb and then negatively stained using 4% ammonium molybdate (Sigma Aldrich, St. Louis, MO, USA) for 2 min. The excess of liquid was removed using filter paper (Whatman, Munich, Germany). The grids were dried for 5 min, then observed at 80 kv using transmission electron microscope JEM–1400 TEM (JEOL, Peabody, MA, USA) at the Veterinary Sciences Division AFBI, Belfast. 

### 2.4. Screening Bacteriophages against Soft Rot Bacteria Species for Cocktail Formulation

Four isolated bacteriophages were tested for virulence using a spotting assay and an overlay assay against 12 bacterial isolates that were isolated in Northern Ireland in the years 2016–2017 from soft rot onion tissue.

For the spotting assay, a total volume of 250 μL of the liquid bacterial culture (*ca*. 10^8^ CFU/mL) was inoculated into TA (5 mL, 37 °C). After gentle vortexing of this mixture, it was poured into prepared NA (CM0003, Oxoid, Basingstoke, UK) plates and allowed to solidify at room temperature for 30 min to produce bacterial lawns. Phage lysate (20 µL, ca. 10^8^ PFU/mL) was spotted using a pipette onto the TA layer, and left to dry at room temperature for 30 min following incubation at 25 °Cfor 12 h and examination for inhibition zones.

For the overlay assay, phage lysate dilutions (ten–fold) (100 μL) were mixed with 250 μL of each bacteria isolate (Table S1) and then incubated for 20 min in 25 °C. The obtained ten-fold dilutions were combined with TA (5 mL, 37 °C), then poured into NA (CM003, Oxoid, Basingstoke, UK). The medium was allowed to solidify for 30 min at room temperature, after which the plates were incubated at 25 °C for 24 h and then examined for plaque formation.

The efficiency of plating (EOP) of tested bacteriophages in overlay and spotting assays on each bacteria isolate was determined as the ratio of the titre of the phage on a given cell line/titre of phage on a maximum cell line calculated in PFU/mL after plating [22]. 

### 2.5. DNA Extraction, Purification and DNA Sequencing 

The bacteriophage lysates were filter–purified using a 10 mL syringe barrel fitted with a 0.22 µm filter Millex^®^ GV filter unit (Merck, Darmstadt, Germany) and stored in a 30 mL Sterilin^®^ universal container (Thermo Fisher Scientific, Waltham, MA, USA) at 4 °C prior to use. HTS was performed using the MiSeq™ sequencer (Illumina, San Diego, CA, USA) with v2 2 × 250 sequencing reagents (Illumina, San Diego, CA, USA) following the manufacturer’s instructions for denaturation of a 2 nM library at AFBI Veterinary Sciences Division, Belfast with the modification to the method described previously [23]. 

### 2.6. Genomic and Phylogenetic Analysis 

The obtained fastaq raw reads of each bacteriophage (the forward and reverse strands) were paired into one single read list. The quality was enhanced by trimming off the low-quality reads using BBDuk tool, errors corrected, normalized using BBnorm, chimeric and duplicate reads were removed using tools in Geneious Prime version 2019.1.3 (Biomatters Ltd., Auckland, New Zealand). Corrected sequences were assembled using de novo assembler and placed into scaffolds. Assembled genomes were compared with bacteriophages sequences available in GenBank (National Centre of Biotechnology Information, Bethesda, MD, USA) using blastn tool in Geneious Prime version 2019.1.3 (Biomatters Ltd., Auckland, New Zealand) software. Obtained bacteriophage genomes (partial and complete) were deposited in GenBank under accession numbers MN518139, MN509793, MN692199 and MN692200.

Phylogenetic analysis was performed to identify the taxonomic position of the bacteriophage genomes within *Caudovirales* order. Multiple alignments of four bacteriophages were performed using CLC Genomics Workbench 9.5.4 (Qiagen, Redwood City, CA, USA) with bacteriophage’s reference capsid proteins available in GenBank including following genera of *Siphoviridae: Chivirus* (ATW62400.1, AZV00099.1, AXY84874.1), *Nonagvirus* (YP_009219975.1, YP_009216943.1), *Myunavirus* (ATS94104.1, YP_006906393.1), *Seuratvirus* (YP_009196799.1, YP_0091511949.1) and two unclassified genera (AZS06248.1) and (AZS06320.1). For two obtained *Podoviridae* bacteriophages the genomes were aligned with members of *Phimunavirus* (KT2401861.1, NC_031068), *Phikmvvirus* (ABY71003.1, YP_033345495.1), *Teseptimavirus* (KY124276.2, KY250035.1), *N4-like virus* (KY549659.1, KY514264.1 and NC_021772.1) and two further unclassified phages (NC_019911.2 and MK053931.1). 

Bacteriophage genomes were mapped and open reading frames (ORFs) were predicted using SnapGene^®^ (GSL Biotech LLC., Chicago, IL, USA). Further analysis of predicted ORFs gene products was conducted with the blastp (protein-protein BLAST) [24] tool using SnapGene® (GSL Biotech LLC., Chicago, IL, USA). The obtained genomes were also annotated for number of subsystems and coding sequences (CDS) with Rapid Annotations using Subsystems Technology (RAST) annotation scheme RASTtk pipeline version 2.0 [25] with threshold repeat region SEED at a minimum 95% identity and minimum length 100 bp. CDS were predicted using Glimmer 3.0 and Prodigal with RAST [25]. The analysis of conserved domains in bacteriophage genomes was performed using conserved domains database (CDD) [26]. The presence of transfer RNAs were screened using rRNA–SEED and tRNA–trnascan with the use of RASTtk [25]. Detection of potential acquired antimicrobial resistance genes present in bacteriophage genomes was performed using ResFinder 3.2 [27] with identity threshold at 90% and minimum coverage 60%. Screening for encoding virulence genes of human bacteria: *Listeria*, *Staphylococcus aureus*, *Escherichia coli*, *Enterococcus* and toxins was performed using Virulence Finder 2.0 [28] with identity threshold 90% and minimum coverage 60%. Identification of genes involved in mycotoxin synthesis was performed using ToxFinder 1.0 [29] with the default setting option.

### 2.7. Phage Cocktail Used in Field Trials

For the cocktail of non-formulated bacteriophages, four lysates (φMA11, φMA12, φMA13 and φMA14), were mixed with a ratio 1:1:1:1, with each phage lysate adjusted to be ca. 10^8^ PFU/mL. The phage cocktail was stored at 4 °C until use. Phage cocktail for field trial application was formulated through enriching a volume of 1 mL of the formulated phage cocktail and 500 µL of liquid bacterial suspension (OD A_600_ = 0.2 or approximately ca. 10^8^ CFU/mL) in 800 mL of NB and made up to 1 L with sterile water. The mixture was incubated overnight at 25 °C with agitation (200 rpm). After overnight incubation, the phage cocktail was separated from the bacterial suspension by filtration using a Millipore Express TM^®^ Plus 0.22 µm filter (Merck, Darmstadt, Germany) and collected into a Millipore filter receiver flask (Merck, Darmstadt, Germany) connected to a general-purpose vacuum pump. The concentration of phage cocktail for field trial application was standardized to be ca. 10^6^–10^8^ PFU/mL. Phage cocktail was stored at 4 °C prior to field application.

### 2.8. Planting Material

Stuttgarter cultivar onion bulb seeds of UK origin were sourced from a commercial supplier in years 2016–2018 and planted in April each year. Onion bulbs were stored at 5 °C prior to use in the field trial.

### 2.9. Treatments and Evaluation Methods Used in Field Trials

Two application methods (immersion, and spraying) of phage cocktail on onions in the years 2016–2018 were used in the field trials with three treatments (spraying _(T1),_ spraying _(T2)_, immersion _(T3)_) and three controls (untreated _(C1),_ negative _(C2)_, positive _(C3)_) tested in three locations in Northern Ireland (Table 1). In each year, trials were performed in different locations to avoid transmission of pathogens and phages (Table 1).

During three years of trials, a 20 × 6 block statistical design was used (including 20 onions/plot, 120 onions/treatment, 120 onions/block). 

From 2016–2018, differences in emergence, soft rot frequency and yield after harvest (mass of bulbs and foliage) were recorded between the five treatments. Soft rot frequency was confirmed due to *Pectobacterium* spp. through isolation on CVP media [30], real-time PCR or/and *recA* gene sequencing using the method previously reported [20] (Table S1). Emergence was calculated as the number of plants planted that were growing approximately 7–8 weeks after planting (out of a total of 120 onions/treatment).

### 2.10. Persistence of Phage Cocktail Treated Tubers in Field Trial 2016

To supplement knowledge about bacteriophage persistence in treated onions for extended periods of time, onions harvested in 2016 were planted in the field trial in 2017 and assessed for emergence, soft rot frequency and yield. A 5 x 6 blocks statistical design was tested (including 5 onions/plot, 30 onions/treatment, 30 onions/block). Emergence was calculated as detailed previously (out of a total of 30 onions/treatment).

### 2.11. Statistical Analysis

Statistical analysis was carried out using GenStat^®^ release 16.2 software (VSN International Ltd, Hemel Hempstead, UK). One-way analysis of variance (ANOVA) was used to compare efficacy of the treatments on yield, emergence and soft rot frequency on onions. Multiple comparisons were performed using Fisher’s least significant difference (LSD).

## 3. Results

### 3.1. Isolation, Purification and Identification of Bacteriophages

**Figure 1:**
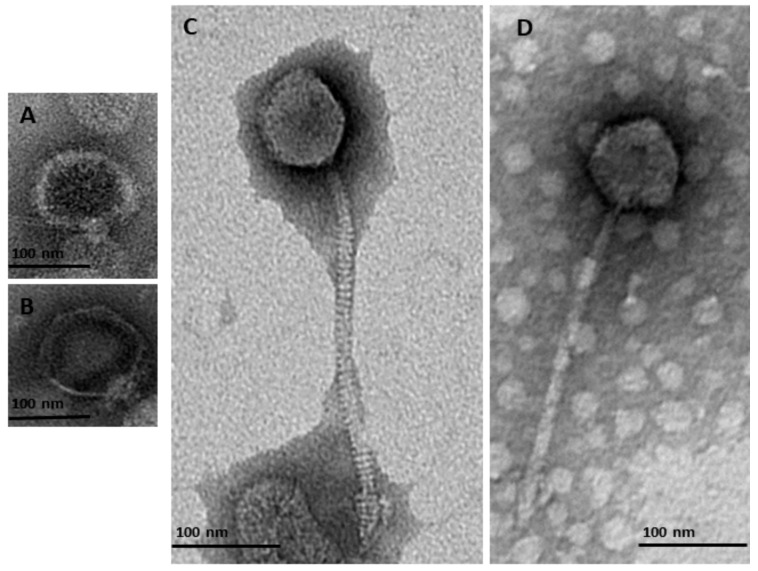
Transmission electron micrographs of negatively stained bacteriophages identified in this study from the order *Caudovirales* belonging to two families of *Podoviridae* (**A**) φMA13; (**B**) φMA14; and *Siphoviridae* (**C**) φMA12 (**D**) φMA11.

Characterization using TEM of purified and titrated bacteriophage particles revealed four bacteriophages belonging to two families of the order *Caudovirales*: *Podoviridae* and *Siphoviridae*. Two of the detected bacteriophages belonged to the *Podoviridae* family (Figure 1A,B and Table 2). These had a short non-contractile tail (φMA13) and a tail 11.22 nm length (φMA14) (Table 2). Two bacteriophages were characterized as members of the *Siphoviridae* family (Figure 1C,D) and had icosahedral heads (50.1–58.7 nm × 42.1–48.78 nm) and flexible tails (223–227 nm) (Table 2).

### 3.2. Genomic Characterization of Bacteriophages

**Figure 2:**
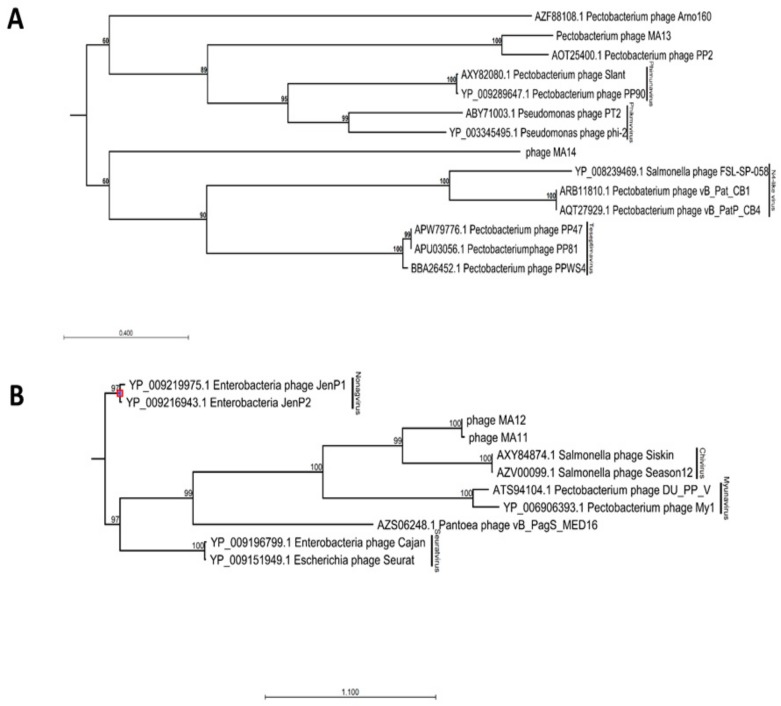
Maximum-likelihood phylogenetic analysis of major capsid proteins of phages isolated in this study constructed using a neighbor–joining method with protein substitution model Dayhoff (PAM) using CLC Genomic Workbench 9.5.4. (**A**) Members of *Podoviridae* family phages. (**B**) Members of *Siphoviridae* family phages. For phages obtained from GenBank capsid proteins accession numbers followed by phages names; phages isolated in this study: phage MA11, phage MA12, phage MA13 and phage MA14. Bootstrap probabilities values < 50% were collapsed.

BLAST analysis of φMA13 and φMA14 bacteriophages revealed these have a taxonomic rank of *Podoviridae* family of unclassified *Autographivirinae* subfamily (Table 2). In phylogenetic analysis of major capsid proteins phages φMA13 and φMA14 grouped in two separate clusters. Phage φMA14 clustered separately in the group of several unclassified bacteriophages (*Pectobacterium* phage Arno160 and *Yersinia* phage 80–18) (Figure 2A). Bacteriophage φMA14 grouped in separate cluster with unclassified subfamily *Pectobacterium* phage PP2 (Figure 2A). 

BLAST analysis [24] of φMA11 and φMA12 bacteriophages revealed the highest similarity to *Siphoviridae* family of *Chivirus* genera (Table 1). Phages φMA11 and φMA12 grouped in two main clusters with *Siphoviridae* family bacteriophages obtained from GenBank in phylogenetic analysis of major capsid proteins. Separate sub–clusters grouped *Salmonella Chivirus* phages (KFS–SEI, Season12 and Siskin) and phages φMA11, φMA12 distinct from other subfamilies (Figure 2B).

For assembled genomes of *Siphoviridae* bacteriophages ORFs were identified to be 55 (φMA11), 61 (φMA12) and 52 for *Podoviridae* φMA13. The conserved domains were identified in 32 ORFs of φMA11, 30 of φMA12 and 18 of φMA13 (Table S2).

**Figure 3:**
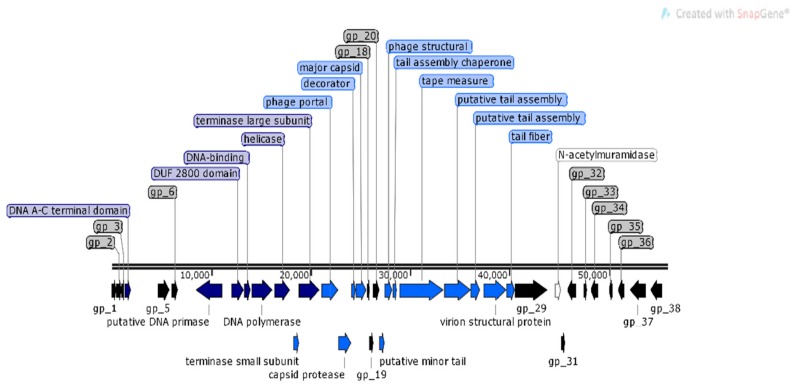
Structural and functional annotation map of φMA11 bacteriophage (55,830 bp) for 55 open reading frames (ORFs) encoding proteins. ORFs coding for the following proteins: hypothetical (black), structural proteins (light blue), proteins for phage replication and lifecycle (dark blue), lysis (white).

Analysis of φMA11 genome revealed the presence of 17 orphan genes (ORFans) with no reliable identity found in GenBank database (*E*–values > 0.001), 20 ORFs for which functions were predicted and 18 hypothetical proteins (Figure 3). 

**Figure 4:**
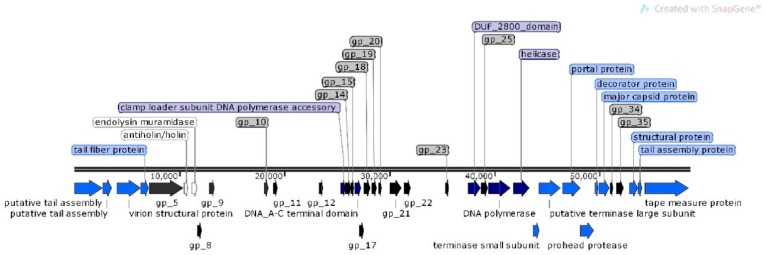
Structural and functional annotation map of φMA12 bacteriophage (58,735 bp) for 60 ORFs encoding proteins. ORFs coding for the following proteins: hypothetical (black), structural proteins (light blue), proteins for phage replication and lifecycle (dark blue), lysis (white).

For φMA12 genome nine ORFans were identified with (*E*–values > 0.001), 22 ORFs with predicted functions and 29 hypothetical proteins (Figure 4). 

For bacteriophage φMA13 from 51 identified ORFs, 20 have predicted functions, 11 hypothetical proteins and 20 (ORFans) with no reliable functions identified.

**Figure 5:**
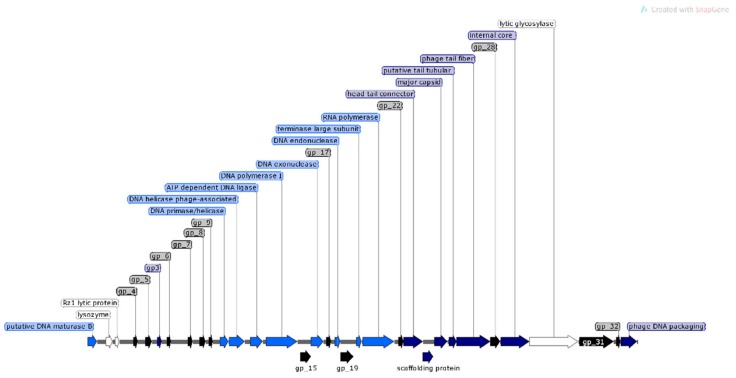
Structural and functional annotation map of φMA13 bacteriophage (42,464 bp) for 51 ORFs encoding proteins. ORFs coding for the following proteins: hypothetical (black), structural proteins (light blue), proteins for phage replication and lifecycle (dark blue), lysis (white).

The analysis of φMA11, φMA12 and φMA13 genomes revealed clusters of proteins involved in DNA replication, recombination, repair and suppression of the host such as: DNA polymerases (φMA11; φMA12; φMA13), DNA helicases (φMA11; φMA12; φMA13), ssDNA–binding proteins (φMA11; φMA12), terminases small and large subunits (φMA11, φMA12, φMA13), DNA A-C terminal domains (φMA11; φMA12) and DUF 2800 domain (MA11; φMA12) (Figure 3, Figure 4 and Figure 5). Clusters of additional proteins involved in these processes has been predicted in *Podoviridae* phage φMA13 such as putative DNA maturase, protein gp3, ATP–dependent DNA ligase, DNA exonuclease, DNA endonuclease and RNA polymerase (Figure 5).

Bacteriophages φMA11 and φMA12 genomes encode endolysin and holin proteins involved in lysis process: N-acetylmuramidase (DUF 3380 superfamily protein cl13324) (φMA11; φMA12) and antiholin/holin protein (φMA12) (Figure 3 and Figure 4). Bacteriophage φMA13 encodes three proteins related to lysis module such as: lysozyme, Rz1 lytic protein and lytic glycosylase (Figure 5). 

Gene products predicted to be involved in virion structure and assembly were organized as a discrete module involving phage portal (φMA11; φMA12), major capsid (φMA11; φMA12; MA13), phage structural protein (φMA11; φMA12), capsid protease (φMA11), prohead protease (φMA12), decorator (φMA11; φMA12), tail assembly chaperone (φMA11), tape measure (φMA11; φMA12), putative tail assembly (φMA11), tail fiber (φMA11; MA13), and virion structural proteins (φMA11) (Figure 3, Figure 4 and Figure 5). For bacteriophage φMA13 four further structural proteins has been predicted including: head tail connector, putative tail tubular, internal core and phage DNA packaging (Figure 5). 

RASTtk pipeline analysis using mRNA and tRNAscan-SE did not reveal the presence of RNAs in bacteriophages genomes (Tables S3 and S4). Virulence Finder, ResFinder 3.1 and ToxFinder revealed no genes that encode antibiotic resistance, toxins or taking a part in synthesis of mycotoxins (Table S3).

### 3.3. Phage Cocktail Formulation 

Clear plaque formation in overlay assays and/or lysis of biofilm in spot assays revealed that all phages could lyse *Pectobacterium* spp. isolates, while φMA12 and φMA14 lysed *Pantoea* spp. isolates (Table 3). 

### 3.4. Evaluation of Efficacy of the Phage Cocktail in Vivo

A protective effect against soft rot development was observed in all three years of field trials that evaluated phage cocktail-treated onions. Plants treated through spraying treatments _(T1)_ and, _(T2)_, and the immersion treatment _(T3)_ had significantly fewer soft rot symptoms in comparison to controls (Table 4). 

In the 2016 field trial, there was no significant influence of the phage cocktail through any of the three treatments on the emergence or yield (mass of bulbs, or mass of foliage) after harvest in comparison to the untreated control as determined by one-way-ANOVA. There were significantly fewer (*p* < 0.05) soft rot symptoms in the phage cocktail treated onions, irrespective of application method (both spraying methods and the immersion method) in comparison to the untreated control according to Fisher’s LSD test (Table 4). 

There was no significant difference between the emergence of phage cocktail treated plants and untreated plants (control) in the 2017 field trial (Table 4). In comparison to untreated onions, there was a significantly higher onion yield (i.e., higher bulb and foliage mass) in the immersion treatment compared with the positive control (Table 4). Significant differences were obtained from harvested onions in terms of the incidence of soft rot (*p* < 0.05), with less soft rot in the spraying (_(T1)_, _(T2)_) and immersion _(T3)_ treatments than in the untreated onions and positive control plants according to a Fisher’s LSD test. 

For the field trial in 2018, significant differences (*p* < 0.001) were recorded in terms of emergence, yield (of bulbs and foliage) and soft rot symptom frequency between treatments (Table 4). A Fisher’s LSD test showed a significantly higher numbers of plants for all treatments when compared to the untreated and positive control (*p* < 0.05). The yield (mass) of onions bulbs using the spraying _(T2)_ and immersion _(T3)_ treatments were not significantly different from the untreated plants (Table 4). Plants treated with phage cocktail showed significantly fewer soft rot disease symptoms in comparison to the positive or negative controls (Table 4).

### 3.5. Persistence Trial of Bacteriophages in Phage Cocktail Treated Onions 

The persistence experiment in 2017 revealed a significant difference between emergence of phage cocktail treated plants (treated in 2016) using both application methods and untreated control plants (Table 5). Significant differences were recorded between soft rot frequency of treated plants with phage cocktail for all three treatments and untreated control plants by ANOVA (*p* < 0.05). Soil drench and foliage application treatments with the phage cocktail significantly increased bulb and foliage mass compared with the untreated control (Table 5).

## 4. Discussion

SRP (mainly *Pectobacterium* and *Dickeya*) species are the main causes of soft rot disease [31], and therefore this study focused on isolation of prospective phages to target *Pectobacterium* spp. (i.e., *P. c.* subsp. *carotovorum*). Interestingly, two of the isolated bacterial strains from onion soft rot tissue were identified as *Pantoea* spp., with one showing the most similarity by BLAST to *Pantoea agglomerans* (*Pa. agglomerans*) and the second to *Pantoea dispersa*. *Pa. agglomerans* is known to be endophytic in plants [32], but has been reported to cause soft rot of onions previously [33,34] Therefore, four phages were tested against these two isolated *Pantoea* spp., and could also serve as an indicator of the ability of the phages to infect a broad range of other closely related plant pathogenic bacteria. The vast majority of SRP bacteriophages (infecting *Pectobacterium* and *Dickeya*) tested for biocontrol applications belong to *Podoviridae* and *Myoviridae* families [35], with the *Podoviridae* bacteriophages infecting strains of *P. c.* subsp. *carotovorum* [12], *P. atrosepticum* [17,36], *Pectobacterium parmentieri* [37,38], and *Dickeya solani* (*D. solani*) [39]. One of the bacteriophages isolated and tested belongs to the *Podoviridae* family, and has a host range that includes *P. c*. subsp. *carotovorum* and *Pantoea* sp. Thus far, two bacteriophage phiKMV-like viruses of *Podoviridae* family have been characterized as infecting *Pa. agglomerans* strains of potato origin [40,41].

In 2017, a new genus of *Autographivirinae* subfamily phages was proposed and reported as having potential for biocontrol applications [42]. The phage PP2 infecting *P. c*. subsp. *carotovorum* isolated from Chinese cabbage showed significant genetic distinction to other bacteriophages of *Autographivirinae* subfamilies [42]. In this study, bacteriophage φMA13 showed high similarity to phage PP2 in a phylogenetic analysis based on the genes related to major capsid protein and genome composition. Similar to previous findings of *Podoviridae* bacteriophages virulent against SRP [12,17,37,38], phage φMA13 contains genes encoding proteins involved in bacteria cell lysis and host suppression, and lacked proteins encoding for toxins or bacterial resistance. This bacteriophage could therefore be safely used for phage cocktail formulation.

*Siphoviridae* is the most abundant bacteriophage family within the *Caudovirales* order, which infects a broad range of Enterobacteriaceae species such as *E. coli*, *Serratia*, *Bacillus*, *Pseudomonas*, and *Klebsiella* spp. [41]. Although the virulence of this bacteriophage family against SRP or *Pantoea* spp. has not been frequently described in scientific literature, the family has been reported to infect *P. c.* subsp. *carotovorum* such as φMy1 [43], *Pectobacterium* phage DU_PP_V [44], *D. solani* (φCIM1) [39] and *Pa. agglomerans* [41]. It has been indicated that some *Siphoviridae* bacteriophages could transmit generalized transduction which might be a threat for phage therapy due to resistance gene transfer between bacteria and phages [45,46]. Genomic analysis of two of the examined *Siphoviridae* phages did not indicate bacterial host homologs mRNA/tRNA (*E* < 0.001) that could indicate lysogenic lifecycle and transduction abilities. Moreover, conserved domain analysis indicated that lysis, replication and suppression of the host modules are well conserved within phages φMA11 and φMA12 (Table S3). Bacteriophage φMA11 was the most virulent against several *P. carotovorum* isolates tested in this study and phage φMA12 has the broad host range within *P. carotovorum* and *Pantoea* sp. Interestingly, the protein DUF 2800 domain of both φMA11 and φMA12 bacteriophages was predicted by CDD analysis to encode a sequence of *Cas4* protein (Cas4_I-A_I-B_I-C_I-D_II-B super family cl00641) (Table S3) belonging to a clustered regularly interspaced short palindromic repeats-CRISPR associated system (CRISPR-Cas). CRISPR-Cas system is a well explored acquired immunity defense system of bacteria and archaea against other genetic materials such as plasmids and bacteriophages, which prevent against infection [47]. However, bacteriophages might evade immunity and use anti-CRISPR-Cas proteins such as *Cas4* protein to infect bacteria [48,49].

The persistence of bacteriophage treatments in crops over multiple growing seasons has not been explored in the field, though evidence exists regarding the persistence of SRP bacteriophages on potato tubers or leaves in vitro [13]. In this study, the efficacy of the phage cocktail appeared to be carried over to the year after treatment. These findings might indicate that bacteriophages like other viruses (i.e., potato virus Y) can be translocated and survive within plants over time [50]. The evidence presented here suggests that these findings should be investigated further.

Phage cocktail formulations containing several phages have been found to protect plants against the plant pathogenic bacterial species *Erwinia amylovora*, *Xanthomonas campestris* pv. *vesicatoria*, *Xanthomonas axonopodis* pv. *citri*, *Xanthomonas citrumelo*, *Ralstonia pseudosolanacearum* and *Dickeya* spp. in bioassay experiments [51]. By contrast, a formulation of a single bacteriophage efficacious against *D. solani* has been reported with limited success to control soft rot disease in field conditions [11]. In this study, in vivo experiments under field conditions showed that there is potential to use a phage cocktail to protect onions against the symptoms of soft rot (most likely caused by *P. c.* subsp. *carotovorum*) using spraying _(T2)_, which was efficacious in three growing seasons. The other two treatments: spraying _(T2)_ and immersion _(T3)_, were efficacious in two years of trials. Moreover, there was a significant difference in 2018 between spraying from the first day of planting _(T1)_ and spraying only from four weeks after planting _(T2)._ As inconsistency of biological control formulations has been shown previously, with a wide range of factors thought to influence this inconsistency [14], including variability of treatments caused by bacteriophages and their interaction in the cocktail (i.e. through synergy) [52]. Four of the fifteen strains of *Pectobacterium* could not be controlled in vitro using any of the bacteriophages tested (Table 3). All of these resistant strains were from Co. Antrim. Despite this finding, results from the field trials showed that the bacteriophage cocktail was efficacious at controlling soft rot of onions. This result supports previous studies [2,19] cautioning on extrapolating results from in-vitro studies to in field conditions. This study did not investigate synergy or inter-bacteriophage effects, and therefore it is difficult to assess which bacteriophages act as potential donors (enzymes producing phages which improve adsorption rate of other phages) with their recipient (phage which is benefiting from the enzyme) bacteriophages in the cocktail [52]. Therefore, these treatments should be further optimized (e.g., by calculating optimal concentration of each bacteriophage, and testing the optimal intensity of applications and synergy) during future investigations.

The spraying _(T2)_ method was the most effective during three growing seasons; however, optimization of the immersion _(T3)_ treatment would be the most beneficial from a practical point of view for the growers as this could be used for pre-treatment of onion seeds before planting reducing the need for specialist applications in the field. Further efficiencies could be made to the phage production process by replacing the nutrient broth stage for building up sufficient numbers of phages and purification.

This study was not comprehensive, as it did not carry out tests of phage kinetics in vitro (suppression and stability) and focused more on the ability of the phages to control soft rot in field settings. Therefore, further investigations are needed to examine the interaction of phages with the host using killing curves and stability experiments. Future experiments should also include a greater range of test bacterial isolates which cause disease in onions (e.g. *Dickeya* spp., *Xanthomonas* spp. and *Pseudomonas* spp.). The use of standard control isolates in overlay assays and bioassays, and more replicated laboratory and field studies would help to further prove the efficacy of these phages for control of a broader range of bacterial isolates pathogenic to onion. Further studies are also needed to investigate the epidemiology and pathogenicity of soft rot in onions for detection of all pathogens known to cause this disease in Northern Ireland. 

## 5. Conclusions

This study provides the first evidence of the use of *Siphoviridae* and *Podoviridae* bacteriophages as a plant protection biocontrol cocktail formulation to protect onions against natural bacterial soft rot infection under field conditions. As a significant protective effect against natural soft rot infection has been observed, future work should seek to further optimize and test the phage cocktail under laboratory and field conditions.

## Figures and Tables

**Table 1:** Details of field trials performed in years 2016–2018.

Year	Location	Number of Applications per Treatment × Volume [L] /Plot					
		Spraying (T1) ^1^	Spraying (T2) ^2^	Immersion (T3) ^3^	Untreated (C1) ^4^	Negative (C2) ^5^	Positive (C3) ^6^
2016	Belfast, Co. Antrim, NI	4 × 1	4 × 1	1 × 2	NT	1 × 2	1 × 2
2017	Crossnacreevy, Co. Down, NI	4 × 1	4 × 1	1 × 2	NT	1 × 2	1 × 2
2018	Loughgall, Co. Armagh, NI	4 × 1	4 × 1	1 × 2	NT	1 × 2	1 × 2

^1^ For spraying _(T1)_, planted onions were sprayed with the phage cocktail starting from the first day of planting, and this continued once per week for the next four weeks at an application rate of 1 L/plot. ^2^ Spraying _(T2)_ treatment involved spraying onions with phage cocktail two weeks after planting at an application rate of 1 L/plot, and this continued once per week for the four following weeks. Spraying was performed using a Knapsack spraying pump directly into the soil on rows with planted material (spraying _T1_) or visible onion foliage (spraying _T2_). ^3^ Treatment immersion _(T3)_ was achieved by submerging onion bulb seeds in 2 L of phage cocktail for 24 h before planting. ^4^ Untreated _(C1)_ control plots received no phage or bacterial treatments throughout the experiment. ^5^ For negative control _(C2)_ onions were immersed in 2 L of NB and left to soak for 24 h before planting. ^6^ As a positive control _(C3)_, onions were immersed in 2 L of bacteria suspension ca. 10^8^ CFU/mL. NT—not treated.

**Table 2:** Characteristics of bacteriophages isolated in this study.

Accession No.	Phage ID	Morphological Characteristics ^a^		Order ^a,b^	Family ^a,b^	Subfamily/Genus ^b^	Id ^b^ (%)	Genome Size (bp)
		Head (nm)	Tail (nm)					
MN518139	φMA11	50.1 × 42.1	223.4	*Caudovirales*	*Siphoviridae*	*Chivirus*	70	55,830
MN692199	φMA12	58.7 × 48.7	227.9	*Caudovirales*	*Siphoviridae*	*Chivirus*	65	58,573
MN509793	φMA13	67.1 × 72.1	nm	*Caudovirales*	*Podoviridae*	*Autographivirinae*	76	42,464
MN692200	φMA14	57.3 × 55.1	11.22	*Caudovirales*	*Podoviridae*	*Autographivirinae*	71	10,019

^a^ Features identified by TEM; ^b^ Features annotated by blastp is % identity of nucleotide sequence with the closest phage by nucleotide sequence; nm-not measured tail.

**Table 3:** Host range of bacteriophages used in this study.

Isolate Id	County Location	Year of Isolation	EOP of Bacteriophages ^1^			
			φMA11	φMA12	φMA13	φMA14
*Pectobacterium* spp.						
OM/Z-1/15	Armagh	2015	(1)	(1)	(1)	-
OM/Z-4/15	Armagh	2015	(1)	-	-	-
OM/Z-5/15	Armagh	2015	1 *	0.01	0.001 *	0.04
OM/Z-1/10/15	Armagh	2015	-	-	-	0.5
OM/Z-2/10/15	Armagh	2015	-	-	-	0.1
05A/16	Down	2016	(1)	-	(1)	(1)
05B/16	Down	2016	-	-	(1)	(1)
06/16-1	Down	2016	(1)	(1)	(1)	(1)
O7/16	Antrim	2016	-	-	-	-
O7B/16	Antrim	2016	-	-	-	-
O17A/16	Antrim	2016	-	-	-	-
O12/16	Antrim	2016	-	-	-	-
O17B/16	Antrim	2016	0.003	-	-	-
O7C/16	Antrim	2016	0.01	0.01	-	-
015/16	Antrim	2016	-	0.1	-	-
*Pantoea* spp.						
O21/16	Antrim	2016	-	1^*^	-	-
O21B/16	Antrim	2016	-	-	-	1*

^1^ Efficiency of plating (EOP) of bacteriophages tested in this study in overlay assay determined as the titre of the phage on a given cell line/titre of phage on a maximum cell line. Asterisks (*) indicated denominator cell line; ‘-‘ no plaque obtained by overlay and spotting assays. Value in brackets indicated titer obtained in spotting assay.

**Table 4:** Mean values of onions treatments assessed in field trials 2016–2018.

Treatment	Emergence ^4^ (%)			Bulbs Mass ^5^ (g)			Foliage Mass ^6^ (g)			Disease ^7^ (%)		
Year	2016 ^1^	2017 ^2^	2018 ^3^	2016 ^1^	2017 ^2^	2018 ^3^	2016 ^1^	2017 ^2^	2018 ^3^	2016 ^1^	2017 ^2^	2018 ^3^
Means												
Spraying _(T1)_^8^	54.2a	91.7a	86.7a	642a	2270b	2040a	282a	1312b	881ab	3.3a	0a	1.7b
Spraying _(T2)_^9^	72.5a	95.0a	70.8b	819a	1988bc	1242b	481a	1526ab	458bc	3.3a	0a	0c
Immersion _(T3)_^10^	63.3a	90.8a	82.5ab	466a	3056a	1399b	222a	1696a	585ab	3.3a	0a	2.5b
Untreated _(C1)_^11^	54.2a	85.0a	55.0c	642a	2383b	702c	245a	624c	301c	18.3b	10.0b	5.0b
Negative _(C2)_^12^	59.2a	90.0a	78.3ab	561a	1608c	1587ab	342a	795c	900a	15.8b	12.5b	10.8a
Positive _(C3)_^13^	NT	39.2b	25.3d	NT	324d	313d	NT	249d	203c	NT	13.3b	1.8b

^1^ Field trial performed in Belfast, Co. Antrim, Northern Ireland, UK; ^2^ Field trial performed in Crossnacreevy, Co. Down, Northern Ireland, UK; ^3^ Field trial performed in Loughgall, Co. Armagh, Northern Ireland, UK; ^4^ Percentage emergence (%Em) = number of plants assessed × 100%/number of plants planted; ^5^ mass of onion bulbs harvested; ^6^ mass of onion foliage harvested; ^7^ percentage of soft rot (%Sr) = number of plants with soft rot symptoms × 100% / total number of plants emerged; ^8^ onions sprayed with phage cocktail from first day of planting for four weeks; ^9^ onions sprayed with phage cocktail after first four weeks; ^10^ onions pre-treated with phage cocktail before planting; ^11^ onions not pre–treated before planting; ^12^ onions pre-treated with NB before planting; ^13^ onions pre–treated with artificial inoculum of bacteria (*ca*. 10^8^ CFU/mL) before planting. Treatment means that do not share the same letter (a, b, c, and d) within each column are significantly different according to Fisher’s Least significant difference at *p* < 0.05; NT—not tested.

**Table 5:** Means of treatments in the persistence trial.

Treatment	Emergence ^5^ (%)	Soft Rot ^6^ (%)	Mass of Bulbs ^7^ (g)	Mass of Foliage ^8^ (g)
Means				
Spraying _(T1)_^1^	93.3 **	3.3 *	749 *	807 *
Spraying _(T2)_^2^	80.0 **	3.3 *	541 *	666 *
Immersion _(T3)_^3^	30	3.3 *	346	314
Control (Untreated) ^4^	36.7	30	314	288

^1^ Onions sprayed with phage cocktail in field trial 2016 starting from first day of planting and continued for the four following weeks at an application rate 1 L/plot and planted and assessed in 2017; ^2^ Onions sprayed with phage cocktail in field trial 2016 starting for four following weeks at an application rate 1 L/plot (approximately 2 weeks after planting) and planted in 2017; ^3^ The onion bulbs immersed in field trial 2016 in 2 L of phage cocktail for 24 h before planting and planted in 2017; ^4^ Untreated onions with phage cocktail and bacteria growing in field trial 2016 and planted in 2017; ^5^ Percentage emergence = number of plants assessed × 100% / number of plants planted; ^6^ Percentage of soft rot = number of plants with soft rot symptoms × 100%/total number of plants tested; ^7^ Mass of onions bulbs harvested; ^8^ Mass of onions foliage harvested. Asterisk indicates a significant difference between the phage-based cocktail formulation treatments and control treatment (untreated) according to Fisher’s least significant difference at *p* < 0.05 (*) and *p* < 0.001 (**).

## Supplementary Materials

The following are available online at https://www.mdpi.com/1999-4915/12/2/150/s1, Table S1: Bacteria isolates used in this study, Table S2: Conserved domains of bacteriophages isolated in this study, Table S3: Features of annotated bacteriophages genomes in this study by RAST, Virulence Finder, Res Finder 3.1 and ToxFinder, Table S4: RAST annotations.
