# Supplementary material for: Genomic Characterization, Formulation and Efficacy in Planta of a Siphoviridae and Podoviridae Protection Cocktail against the Bacterial Plant Pathogens Pectobacterium spp
Source: Viruses. 2020 Jan 28;12(2):150. doi: 10.3390/v12020150 (PMC7077305; doi:10.3390/v12020150)
Supplement: Supplementary file 1 [file viruses-12-00150-s001.zip › Supplementary materials-REVISED 27.1.20/Table S1.pdf]

Table 1. Bacterial strains used in this study.

Isolate Id	Location	Year	Cavity formation on CVP media [30]	Y1 & Y2—*P. atrosepticum* [53]	ECA primers—*P. atrosepticum* [54]	PEC primers—*Pectobacterium* and *Dickeya* spp. [54]	ECH primers-*Dickeya* spp. [54]	RecAF & RecAR—formely *Erwinia* spp. [55]	Y45 & Y46—formely *P. carotovorum* [56]	DNA sequencing[h]	Species identified (Accession number)
O7/16	Co. Antrim	2016	+	nt	+	+	-	+	-	-	*P. atrosepticum*
O7B/16	Co. Antrim	2016	+	nt	+	+	-	+	-	-	*P. atrosepticum*
O17A/16	Co. Antrim	2016	+	nt	+	+	-	+	-	-	*P. atrosepticum*
05A/16	Co. Down	2016	+	-	-	+	-	+	+	-	*Pectobacterium*
05B/16	Co. Down	2016	+	-	-	+	-	+	+	-	*Pectobacterium*
06/16-1	Co. Down	2016	+	-	-	+	-	+	+	-	*Pectobacterium*
OM/Z-1/15	Co. Armagh	2015	+	-	-	+	-	+	+	-	*Pectobacterium*
OM/Z-4/15	Co. Armagh	2015	+	-	-	+	-	+	+	-	*Pectobacterium*
OM/Z-5/15	Co. Armagh	2015	+	-	-	+	-	+	+	+	*P.c.* subsp. *carotovorum* (MN922297)
O12/16	Co. Antrim	2016	+	nt	-	+	-	+	nt	-	*Pectobacterium*
O17B/16	Co. Antrim	2016	+	nt	-	+	-	+	nt	-	*Pectobacterium*
O7C/16	Co. Antrim	2016	+	nt	-	+	-	+	nt	-	*Pectobacterium*
015/16	Co. Antrim	2016	+	nt	-	+	-	+	nt	-	*Pectobacterium*
OM/Z-1/10/15	Co. Armagh	2015	+	-	-	+	-	+	+	-	*Pectobacterium*
OM/Z-2/10/15	Co. Armagh	2015	+	-	-	+	-	+	+	-	*Pectobacterium*
O21/16	Co. Antrim	2016	+	nt	-	+	-	+	nt	+	*Pantoea* sp. (MN692198)
O21B/16	Co. Antrim	2016	+	nt	-	+	-	+	nt	+	*Pantoea* sp. (MN720105)

References

53. Fréchon, D.; Exbrayat, P.; Helias, V.; Hyman, L.J.; Jouan, B.; Llop, P.; Lopez, M.M.; Payet, N.; Pérombelon, M.C.M.; Toth, I.K.; et. al. Evaluation of a PCR kit for the detection of *Erwinia carotovora* subsp. *atroseptica* on potato tubers. *Potato Res.* **1998**, *41*, 163–173.
54. Pritchard, L.; Humphris, S.; Saddler, G.S.; Parkinson, N.M.; Bertrand, V.; Elphinstone, J.G.; Toth, I.K. Detection of phytopathogens of the genus *Dickeya* using a PCR primer prediction pipeline for draft bacterial genome sequences. *Plant Pathol*. **2013**, *62*, 587–596.
55. Waleron, M.; Waleron, K.; Podhajska, A. J.; Łojkowska, E. Genotyping of bacteria belonging to the former *Erwinia* genus by PCR-RFLP analysis of a *recA* gene fragment. *Microbiology*, **2002**, *148*, 583–595.
56. Darasse, A.; Priou, S.; Kotoujansky, A.; Bertheau, Y. PCR and restriction fragment length polymorphism of a *pel* gene as a tool to identify *Erwinia carotovora* in relation to potato diseases. *Appl. Environ. Microbiol*., **1994**, *60*, 1437–1443.
