# Supplementary material for: Genomic Characterization, Formulation and Efficacy in Planta of a Siphoviridae and Podoviridae Protection Cocktail against the Bacterial Plant Pathogens Pectobacterium spp
Source: Viruses. 2020 Jan 28;12(2):150. doi: 10.3390/v12020150 (PMC7077305; doi:10.3390/v12020150)
Supplement: Supplementary file 1 [file viruses-12-00150-s001.zip › Supplementary materials-REVISED 27.1.20/Table S3.pdf]

Table S3. Features of annotated bacteriophages genomes in this study by RAST, Virulence Finder, Res Finder 3.1 and ToxFinder.

	Bacteriophage		
**Feature**	**ϕMA11**	**ϕMA12**	**ϕMA13**
GC content (%)[a]	48.9	51.6	53.4
L50[a]	1	1	1
Number of contigs (with PEGs)[a]	1	1	1
Number of subsystems (genes)[a]	5	5	1
Number of coding sequences[a]	52	55	55
Number of RNAs[a]	0	0	0
Shiga-toxins[b]	0	0	0
Virulence genes[b]	0	0	0
Antibiotic resistance genes[c]	0	0	0
Mycotoxins[d]	0	0	0

[a] Features determinate by RAST; [b]Features determinate by Virulence Finder; [c]Features determinate by ResFinder 3.1 at threshold 90% for identifying genes encoding resistance for: Aminoglycoside, Beta-lactam, Colistin, Fluoroquinolone, Fosfomycin, Glycopeptide, MLS – Macrolide, Lincosamide, Streptogramin B, Nitroimidazole, Oxazolidione, Phenicol, Rifampicin, Sulphonamide, Tetracycline and Trimethoprim; [d]Features identified by ToxFinder.
